# Do early onset and pack-years of smoking increase risk of type II diabetes?

**DOI:** 10.1186/1471-2458-14-178

**Published:** 2014-02-19

**Authors:** Sun Jung Kim, Sun Ha Jee, Jung Mo Nam, Woo Hyun Cho, Jae-Hyun Kim, Eun-Cheol Park

**Affiliations:** 1 Department of Public Health, Yonsei University College of Medicine, 50 Yonsei-ro, Seodaemun-gu, Seoul 120-752, Korea; 2 Institute of Health Services Research, Yonsei University College of Medicine, 50 Yonsei-ro, Seodaemun-gu, Seoul 120-752, Korea; 3 Department of Epidemiology and Health Promotion and Institute for Health Promotion, Graduate School of Public Health, Yonsei University, 50 Yonsei-ro, Seodaemun-gu, Seoul 120-752, Korea; 4 Department of Preventive Medicine, Yonsei University College of Medicine, 50 Yonsei-ro, Seodaemun-gu, Seoul 120-752, Korea

**Keywords:** Early onset of smoking, Pack-years of smoking, Risk of type 2 diabetes

## Abstract

**Background:**

Type II diabetes is not only major public health problem but also heavy fiscal burden to each nation’s health care system around the world. This study aimed to investigate the effect of early onset and pack-years of smoking on type II diabetes risk.

**Methods:**

We used the most recent cross-sectional National Health and Nutrition Examination Survey set of South Korea (2010) and the United States (2009–2010). Participants who were diagnosed with diabetes after age 20 were included (South Korea: n = 7273, 44% male; U.S.: n = 3271, 52% male). Cox proportional models, stratified by sex and country, were used to estimate hazard ratios.

**Results:**

7.1% of South Korean men, 5.5% of South Korean women, 15.5% of U.S. men, and 12.4% of U.S. women had type II diabetes; 40% of South Korean men, 34% of U.S. men, and 21% of U.S. women began smoking before age 20 (57%, 49%, 36% of those who had type II diabetes, respectively). Type II diabetic participants were older and married; have a higher BMI, low income, and less education; lack moderate physical activity, smoked more and earlier compared to those without type II diabetes. Differences in risk factors including life-style behaviors and SES were found in both diabetic and non-diabetic populations. Men who began smoking before age 16 had a higher type II diabetes risk than who never smoked (South Korea: hazard ratio [HR] 2.46, 95% confidence interval [CI] 1.04–5.79; U.S.: HR 1.64, 95% CI 1.01–2.67), as did U.S. men who began smoking between 16 and 20 years (HR 1.58, 95% CI 1.05–2.37). Smoking pack-years were also associated with type II diabetes in U.S. men (HR 1.07, 95% CI 1.01–1.12). In women population, however, associations were not found.

**Conclusions:**

Early onset of smoking increases type II diabetic risk among men in South Korea and the U.S., and type II diabetic risk increases with higher pack-years in U.S. men, however, no associations were found in women population. Underage tobacco policy and education programs are strongly needed in both countries.

## Background

Type II diabetes is a major public health problem around the world. A systematic analysis of a multinational health examination survey with 370 country-years and 2.7 million participants showed that the prevalence of type II diabetes has doubled throughout the world from 1980 to 2008 [1]. This trend will continue until 2025 [2], and making a heavy fiscal burden to each nation’s health care system [3]. In South Korea, the prevalence of type II diabetes has increased from 1% in 1960 to 9–10% in early the 2000s [4,5], and this disease was ranked in the top five causes of death in 2011 [6]. According to a report of the Korean Health Insurance Review & Assessment Service in 2007, healthcare expenditures of adult diabetic patients accounted for approximately one-fifth of all national health insurance reimbursements (approximately 13 billion USD), and type II diabetes-related costs were almost eight times higher in 2005 than in 1995 [7]. The Centers for Disease Control and Prevention in the United States reported that type II diabetes is the seventh leading cause of death, and the associated direct and indirect costs associated with this disease were estimated at 174 billion dollars in 2007 (direct costs: 116 billion dollars; indirect costs: 58 billion dollars) [8].

Studies conducted in worldwide including U.S. reported that risk factors for type II diabetes include not only obesity [9-12], lack of physical activity [11,13,14], poor diet [15,16], life-style and aging [17,18], but also smoking is plausibly associated with incidence of type II diabetes as well [3,19-25]. Smoking increases insulin resistance [19,26] suggesting that current, heavy smoking may greatly increase the risk for type II diabetes [20,27]. In a 2007 meta-analysis of 25 cohort studies, Willi et al. found that active smoking may increase the risk of type II diabetes [28]. Large cohort studies also confirmed this association between smoking and the development of type II diabetes. Cohort studies carried out in South Korea reported that current smokers have a higher risk of type II diabetes and mortality than non-smokers confirming smoking amount as a risk factor [29], and both current and former smokers have a greater risk of type II diabetes than non-smokers, with the risk increasing with the amount of smoking [30]. Furthermore, the cohort studies also reported that factors including obesity, age, BMI, alcohol drinking, and exercise were also risk factors for diabetes as well [29,30]. Another cohort study, the Western New York Health Study, confirmed that smoking was significantly associated with the conversion from normoglycemia to impaired fasting glucose, after adjusting for other risk factors [31]. Moreover, several other prospective studies [3,19,21-25,32] also demonstrated the association between smoking and type II diabetes, including the United States Physicians Study [20] and Nurses’ Health Study [33] in which both former and current smokers had a greater risk for type II diabetes than non-smokers. Regarding the relationship between incidence of type II diabetes and quantification of smoking, previous studies reported that risk increased with pack-years, amount of smoking, and age of onset smoking [20,27,34-36]. Although multiple studies have reported this association, very few studies have addressed the effect of the combination of onset age of smoking and pack-years on incidence of type II diabetes. Many current, former smokers begin smoking during adolescence [37] and those who begin smoking early are more likely to be current smokers [38]*.* Therefore, it is of interest to investigate whether smoking onset age and pack-years are associated with incidence of type II diabetes. Furthermore, it is another interest to investigate differences in risk factors between the both countries.

## Methods

### Study population

This study used secondary datasets obtained from the most recent National Health and Nutrition Examination Survey (NHANES), which is a cross-sectional and nationally representative survey conducted by the Centers for Disease Control and Prevention of both South Korea and the U.S. Survey participants were non-institutionalized civilians in South Korea and the U.S. who were selected using a stratified, multistage, probability-sampling design based on age, sex, and geographical area. This study used sample weights to produce estimates for the entire populations of South Korea and the U.S. Ethics review boards of the institutions conducting the surveys in both countries approved the study, and participants from both countries provided consent. The most recent available completed datasets were the 2010 Korean NHANES (KNHANES) and the 2009–2010 United States NHANES (USNHANES). Inclusion in this study was limited to participants who received a diagnosis of type II diabetes after age 20, which assumed that they had type 2 diabetes. Of the 4,115 men and 4,843 women from the 2010 KNHANES who were eligible for the study, 3,179 men and 4,094 women with complete data were included in the analysis. Of the 5,225 men and 5,312 women from the 2009–2010 USNHANES, 1,713 men and 1,365 women with complete data were included. Many of survey participants in the U.S. were excluded because of missing information, mainly socioeconomic status covariates. Since the dataset used in this study were publicly available and online accessible data, no ethics committee’s approval is required to conduct the study.

### Outcomes

This study identified type II diabetes cases as participants who answered “yes” to the question “Have you ever been told by a doctor that you have diabetes?”, which was an item on both nations’ NHANES. In addition, a physical examination (including collection of a blood specimen) was added to the KNHANES to determine the diabetes status of participants. Based on the results of blood tests, some participants who had not answered “yes” in the previous questionnaire were identified as having diabetes, and all participants who answered “yes” in the previous questionnaire were also identified as having diabetes. However, the additional number of diabetes cases was minimal for both men and women (<20 cases each). The other outcome variable was age at which diabetes was diagnosed, which both nations’ NHANES assessed using the item, “Age when first told you had diabetes”. For diabetes cases that were added because of blood analysis results, this study used the participants’ current age as the age at diagnosis. This study excluded participants who received a diagnosis before age 20 in an attempt to include only those with type II diabetes.

### Main predictors and covariates

Smoking onset age, smoking pack-years is main predictor of this study. We included age, BMI, family income, education level, marital status, alcohol consumption, physical activity, daily energy intake, and race (U.S. only) as covariates. All main predictors and covariates were obtained from participants’ self-reported answers. Main predictors and covariates included in this study were risk factors for type II diabetes based on the previous researches. Participants were stratified according to smoking onset age into the following four groups based on their answers to the questionnaire item “age started smoking cigarettes regularly”: <16 years, 16–20 years, 21–25 years, or >26 years. The relationship between smoking pack-years and type II diabetes was examined among current smokers, ex-smokers, and never-smokers. Because nations have slightly different questions regarding smoking amount and history, this study used the most appropriate items in KNHANES and USNHANES to estimate smoking pack-years.

For South Korean participants, smoking pack-year was calculated as follows.

• Current smokers with diabetes: (average number of cigarettes/day ÷ 20) × (age at diabetes diagnosis − age started smoking regularly)

• Ex-smokers with diabetes: (number of cigarettes/day when smoking ÷ 20) × year of smoking

• Current smokers without diabetes: (average number of cigarettes/day ÷ 20) × (current age − age started smoking regularly)

• Ex-smokers without diabetes: (number of cigarettes/day when smoking ÷ 20) × years of smoking

For U.S participants, smoking pack years were calculated as follows:

• Current smokers with diabetes: (average number of cigarettes/day ÷ 20) × (age at diabetes diagnosis − age started smoking regularly)

• Ex-smokers with diabetes: (number of cigarettes/day at the time of quitting ÷ 20) × (age last smoked cigarettes regularly − age started smoking regularly)

• Current smokers without diabetes: (average number of cigarettes/day ÷ 20) × (age last smoked cigarettes regularly − age started smoking regularly)

• Ex-smokers without diabetes: (number of cigarettes/day at the time of quitting ÷ 20) × (age last smoked cigarettes regularly − age started smoking regularly)

Age, BMI, and daily energy intake were included as continuous variables in the statistical model. Based on census information, U.S. family income was stratified into quartiles (<25%, 25%–50%, 51%–75%, >75%). South Korean data were used as is (original data was collected as quartiles in South Korea). Education level was categorized into four groups: less than elementary school, less than middle school, high school diploma, at least some college. Marital status was defined as married or other (e.g., widowed, divorced). Alcohol consumption was categorized into three groups: not at all, less than once a month, or more than twice a month during the previous 12 months. Level of physical activity was based on participants’ self-report of moderate intensity activity (yes or no) during the previous 30 days. Because South Korea is not diverse in terms of ethnicity, the KHANES does not ask such information. Race (non-Hispanic white, Mexican American, other Hispanic, non-Hispanic black, or other including multiracial) was included only in the analysis of USNHANES.

### Statistical analysis

For all calculations and analysis, SAS 9.3 (SAS institute, Cary, NC) was used, and separate analyses were conducted by sex. Descriptive statistics were generated; means and standard deviations were calculated for continuous variables, and frequencies and relative percentages were calculated for categorical variables. Chi square statistics were performed to identify group differences by nations among same diabetic status (1. Diabetic Korean Men Vs. Diabetic U.S. Men 2. Non-Diabetic Korean Men Vs. Non-Diabetic U.S. Men 3. Diabetic Korean Women Vs. Diabetic U.S. Women 4. Non-Diabetic Korean Women Vs. Non-Diabetic U.S. Women).

We then examined the crude association between smoking onset age and type II diabetes by using Kaplan–Meier curves. To investigate the associations of smoking onset age, pack-years of smoking and other type II diabetes risk factors with the incidence of type II diabetes, Cox proportional hazard models were used to calculate hazard ratios and the corresponding 95% confidence intervals. In this study, a *P*-value less than 0.05 (two-tailed) was considered statistically significant. Because this study was based on data from NHANES, which uses a complex, stratified, multistage probability cluster sampling structure, SAS survey procedures were used in the Cox proportional hazard models. The study also used the NOMCAR option to perform a domain analysis for observations with and without missing values. Additionally, corresponding weight, cluster, and strata statements were used to determine reliable and nationally representative estimates. The underlying time variable for the Cox proportional hazard models was age at type II diabetes diagnosis. In addition to smoking-related variables, other covariates that are commonly accepted risk factors for type II diabetes were chosen.

## Results

Based on the 2010 KNHANES and 2009–2010 USNHANES datasets, we determined that 225 of 3,179 South Korean men (7.1%), 223 of 4,094 South Korean women (5.5%), 265 of 1,713 U.S. men (15.5%), and 193 of 1,558 U.S. women (12.4%) had type II diabetes. Tables 1 and 2 show participant characteristics according to type II diabetes status of both countries by each sex (Table 1: Diabetic Korean Men Vs. Diabetic U.S. Men and Non-Diabetic Korean Men Vs. Non-Diabetic U.S. Men, Table 2: Diabetic Korean Women Vs. Diabetic U.S. Women and Non-Diabetic Korean Women Vs. Non-Diabetic U.S. Women). In all study groups (except South Korean women), diabetic participants were more likely than non-diabetic participants to have started smoking before age 20. Specifically, 40% of South Korean men (57% of those with type II diabetes), 34% of U.S. men (49% of those with type II diabetes), and 21% of U.S. women (36% of those with type II diabetes) started smoking before age 20. South Korean women reported very low frequencies of smoking; only 4% of the participants (and 1% of diabetic participants) started smoking before age 20. Chi-square statistics show that all other variables were significantly different between diabetic men population of both nations except age, moderate intensity activity, and daily energy intake. Among without diabetic men population of both nations, all variables were significantly different (Table 1). Female population also showed similar trend that age, family income, and daily energy intake were not significantly different between diabetic populations of both nations, but all other variables were significantly different among without diabetic population (Table 2). Korean men started smoking early and more likely to be a heavy smoker. Korean also had more alcohol consumption, exercise less in both men and women than U.S. population regardless of their diabetic status. However, overall energy intake (without type II diabetes) and BMI (both with and without type II diabetes) were fairly higher in U.S population (Tables 1 and 2).

**Table 1:** Characteristics of Men participants stratified by diabetes (diagnosed after age 20) using the South Korea NHANES 2010 and the United States NHANES 2009–2010

	***South Korean men***		***P-value of Chi-square***	***U.S. men***		***P-value*** ***of Chi-square***	***P-value of Chi-square***	
	***(n = 3,179, Est. population = 23,681,690)***			***(n = 1,713, Est. population = 84,085,965)***			***Korea Vs. U.S.***	
	***With diabetes***	***Without diabetes***		***With diabetes***	***Without diabetes***		***With diabetes***	***Without diabetes***
**Variables**	***(n = 225, 7.1*%*)***	***(n = 2,954, 92.9*%*)***		***(n = 265, 15.5*%*)***	***(n = 1,448, 84.5*%*)***			
Smoking Onset Age (%)**								
**Never Smoked**	28 (12.4)	1,372 (46.5)	<.0001	107 (40.4)	918 (63.4)	<.0001	<.0001	<.0001
**≥26 years**	21 (9.3)	106 (3.6)		9 (3.4)	21 (1.5)			
**21–25 years**	49 (21.8)	347 (11.8)		19 (7.2)	59 (4.1)			
**16–20 years**	107 (47.6)	1,021 (34.6)		79 (29.8)	302 (20.9)			
**<16 years**	20 (8.9)	108 (3.7)		51 (19.3)	148 (10.2)			
Smoking Pack-years [10 PY]*	2.5 ± 2.2	1.1 ± 1.7	<.0001	1.8 ± 2.8	0.8 ± 2.0	<.0001	0.002	<.0001
Age (Years)*	63.0 ± 11.0	36.2 ± 23.7	<.0001	62.5 ± 11.6	50.2 ± 17.9	<.0001	0.654	<.0001
**BMI (kg/m**^**2**^)*	24.2 ± 3.2	22.1 ± 4.1	<.0001	31.8 ± 6.7	29.1 ± 5.6	<.0001	<.0001	<.0001
Family Income Percentile (%)**								
**<25%**	85 (37.8)	465 (15.7)	<.0001	76 (28.7)	286 (19.8)	0.009	0.029	<.0001
**25%–50%**	47 (20.9)	801 (27.1)		82 (30.9)	392 (27.1)			
**51%–75%**	44 (19.6)	872 (29.5)		59 (22.3)	312 (21.6)			
**>75%**	49 (21.8)	816 (27.6)		48 (18.1)	458 (31.6)			
Education (%)**								
**Less than Elementary School**	67 (29.8)	1,223 (41.4)	<.0001	47 (17.7)	151 (10.4)	<.0001	<.0001	<.0001
**Less than Middle School**	49 (21.8)	344 (11.7)		43 (16.2)	181 (12.5)			
**High School Diploma**	68 (30.2)	696 (23.6)		61 (23.0)	311 (21.5)			
**At least some College**	41 (18.2)	691 (23.4)		114 (43.0)	805 (55.6)			
Marital Status (%)**								
**Married**	201 (89.3)	1,585 (53.7)	<.0001	178 (67.2)	927 (64.0)	<.0001	<.0001	<.0001
**Other**	24 (10.7)	1,369 (46.3)		87 (32.8)	521 (36.0)			
Alcohol Consumption (%)**								
**Not At All**	66 (29.3)	1,216 (41.2)	0.002	88 (33.2)	265 (18.3)	<.0001	<.0001	<.0001
**Less than Once a Month**	43 (19.1)	423 (14.3)		171 (64.5)	1,123 (77.6)			
**More than Twice a Month**	116 (51.6)	1,315 (44.5)		6 (2.3)	60 (4.1)			
**Moderate Intensity Activity (%)**								
**Yes**	70 (31.1)	1015 (34.4)	0.322	88 (33.2)	614 (42.4)	<.0001	0.621	<.0001
**No**	155 (68.9)	1939 (65.6)		177 (66.8)	834 (57.6)			
Daily Energy Intake (Kcal)*	2,087.6 ± 817	2,268.6 ± 967	0.006	2,046.7 ± 1009	2,428.5 ± 991	0.001	0.627	<.0001
Race (%)**								
**Non-Hispanic Whites**				108 (40.8)	769 (53.1)	0.067	-	-
**Mexican Americans**				57 (21.5)	272 (18.8)			
**Other Hispanics**				31 (11.7)	120 (8.3)			
**Non-Hispanic Blacks**				59 (22.3)	221 (15.3)			
**Other Races (Including Multiracial)**				10 (3.8)	66 (4.6)			

**Mean ± SD.

**Frequency (%).

**Table 2:** Characteristics of Women participants stratified by diabetes (diagnosed after age 20) using the South Korea NHANES 2010 and the United States NHANES 2009–2010

	***South Korean women***		***P-value of Chi-square***	***U.S. women***		***P-value of Chi-square***	***P-value of Chi-square***	
	***(n = 4,094, Est. population = 23,193,263)***			***(n = 1,558, Est. population = 78,989,240)***			***Korea Vs. U.S.***	
	***With diabetes***	***Without diabetes***		***With diabetes***	***Without diabetes***		***With diabetes***	***Without diabetes***
**Variables**	***(n = 223, 5.4*%*)***	***(n = 3,871, 94.6*%*)***		***(n = 193, 12.4*%*)***	***(n = 1,365, 87.6*%*)***			
Smoking Onset Age (%)**								
**Never Smoked**	206 (92.4)	3,558 (91.9)	0.002	99 (51.3)	1,038 (76.0)	<.0001	<.0001	<.0001
**≥26 years**	14 (6.3)	101 (2.6)		10 (5.2)	24 (1.8)			
**21–25 years**	1 (0.5)	66 (1.7)		14 (7.3)	48 (3.5)			
**16–20 years**	1 (0.5)	127 (3.3)		47 (24.4)	189 (13.9)			
**<16 years**	1 (0.5)	19 (0.5)		23 (11.9)	66 (4.8)			
Smoking Pack-years [10 PY]*	0.06 ± 0.45	0.04 ± 0.3	0.296	1.2 ± 2.7	0.4 ± 1.2	<.0001	<.0001	<.0001
Age (Years)*	63.5 ± 12.3	38.9 ± 22.2	<.0001	61.8 ± 12.1	48.6 ± 17.8	<.0001	0.46	<.0001
**BMI (kg/m**^**2**^)*	25.0 ± 3.7	22.0 ± 4.1	<.0001	33.8 ± 7.7	29.0 ± 7.2	<.0001	<.0001	<.0001
Family Income Percentile (%)**								
**<25%**	88 (39.5)	679 (17.5)	<.0001	79 (40.9)	337 (24.7)	<.0001	0.576	<.0001
**25%–50%**	62 (27.8)	989 (25.6)		56 (29.0)	341 (25.0)			
**51%–75%**	41 (18.4)	1,134 (29.3)		28 (14.5)	267 (19.6)			
**>75%**	32 (14.4)	1,069 (27.6)		30 (15.5)	420 (30.8)			
Education (%)**								
**Less than Elementary School**	155 (69.5)	1,687 (43.6)	<.0001	27 (14.0)	86 (6.3)	<.0001	<.0001	<.0001
**Less than Middle School**	21 (9.4)	385 (10.0)		47 (24.4)	155 (11.4)			
**High School Diploma**	38 (17.0)	947 (24.5)		38 (19.7)	275 (20.2)			
**At least some College**	9 (4.0)	852 (22.0)		81 (42.0)	849 (62.2)			
Marital Status (%)**								
**Married**	138 (61.9)	2,142 (55.3)	<.0001	90 (46.6)	709 (51.9)	<.0001	<.0001	<.0001
**Other**	85 (38.1)	1,729 (44.7)		103 (53.4)	656 (48.1)			
Alcohol Consumption (%)**								
**Not At All**	128 (57.4)	1,929(49.8)	0.081	92 (47.7)	272 (19.9)	<.0001	<.0001	<.0001
**Less than Once a Month**	59 (26.5)	1,159(29.9)		96 (49.7)	1,062 (77.8)			
**More than Twice a Month**	36 (16.1)	783(20.2)		5 (2.6)	31 (2.3)			
**Moderate Intensity Activity (%)**								
**Yes**	67 (30.0)	1,170 (30.2)	0.955	47 (24.4)	421 (30.8)	<.0001	0.011	<.0001
**No**	156 (70.0)	2,701 (69.8)		146 (75.7)	944 (69.2)			
Daily Energy Intake (Kcal)*	1,603.1 ± 636	1,702.9 ± 658	0.027	1,616.0 ± 634	1,795.0 ± 693	<.0001	0.56	<.0001
Race (%)**								
**Non-Hispanic Whites**				76 (39.4)	739 (54.1)	0.001	-	-
**Mexican Americans**				36 (18.7)	218 (16.0)			
**Other Hispanics**				20 (10.4)	147 (10.8)			
**Non-Hispanic Blacks**				52 (26.9)	205 (15.0)			
**Other Races (Including Multiracial)**				9 (4.7)	56 (4.1)			

**Mean ± SD.

**Frequency (%).

**Figure 1:**
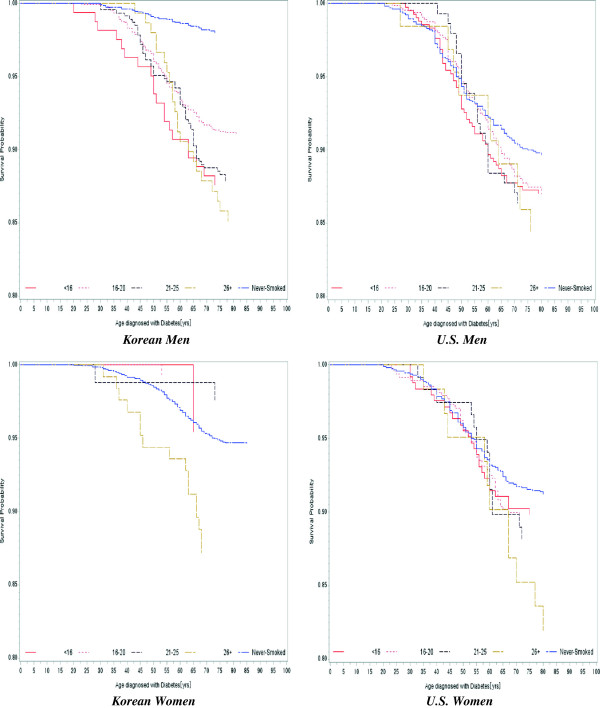
Kaplan–Meier curves for incidence of diabetes according to gender and smoking onset age for South Korea and the United States (using South Korea NHANES 2010 and the United States NHANES 2009–2010).

Figure 1 shows the survival probability for each smoking onset age group (<16 years, 16–20 years, 21–25 years, ≥26 years) and its unadjusted association with type II diabetes. The overall trend demonstrates that smoking increases the risk for type II diabetes relative to non-smokers. The pattern for South Korean women is unusual because of the low case numbers in each smoking onset age group.

Smoking pack-years and BMI were also higher among diabetic participants. Mean age was somewhat higher for diabetic participants, especially among South Korean participants. Type II diabetes appeared to be more prevalent among those who had low income and less education, were married, and did not participate in moderate physical activity; however, results of the relationship between alcohol consumption and type II diabetes were mixed. Daily energy intake was considerably lower in diabetic participants; because most of them had already received a diagnosis of type II diabetes from a physician, diet may be a major concern. Racial disparities were identified in the U.S. population; Mexican Americans, other Hispanics, and non-Hispanic blacks had a higher prevalence of type II diabetes than non-Hispanic whites.

As shown in Table 3, this study assessed the association between smoking onset age and incidence of type II diabetes using Cox proportional hazard models after adjusting for all covariates. This study found that early onset of smoking increased the risk of type II diabetes, especially among men (in both South Korea and the U.S). Among South Korean men, starting smoking before age 16 increased the risk of type II diabetes compared with the reference group who had never smoked (HR 2.46, 95% CI 1.04–5.79). Among U.S. men, starting smoking before age 16 increased the risk of type II diabetes compared with the reference group (HR 1.64, 95% CI 1.01–2.67), as did starting smoking between 16 and 20 years of age (HR 1.58, 95% CI 1.05–2.37). Among U.S. women, this study did not find any statistically significant results; however, the risk of type II diabetes appeared to increase with earlier onset of smoking. This study does not find a significant association between adult onset of smoking (age ≥21 years) and incidence of type II diabetes among men or women of either country. This study found that smoking pack-year was significantly associated with incidence of type II diabetes among U.S. men (HR 1.07, 95% CI 1.01–1.12) but not in the other groups.

**Table 3:** Adjusted hazard ratios for the association between stratified onset age of smoking and diabetes using South Korea NHANES 2010, United States NHANES 2009-2010

	***South Korean men***		***U.S. men***		***South Korean women***		***U.S. women***	
	***Hazard ratio***	***P-value***	***Hazard ratio***	***P-value***	***Hazard ratio***	***P-value***	***Hazard ratio***	***P-value***
	***(95*% *CI)***		***(95*% *CI)***		***(95*% *CI)***		***(95*% *CI)***	
**Smoking Onset Age**								
**Never Smoked**	1.00		1.00		1.00		1.00	
**≥26 years**	1.26 (0.60-2.65)	0.540	1.82 (0.81-4.11)	0.139	1.43 (0.70-2.93)	0.33	1.79 (0.59-5.47)	0.287
**21–25 years**	1.39 (0.71-2.73)	0.340	1.68 (0.79-3.55)	0.163	0.50 (0.06-4.18)	0.519	1.26 (0.48-3.34)	0.622
**16–20 years**	1.54 (0.82-2.88)	0.180	1.58 (1.05-2.37)	0.031	0.10 (0.01-0.84)	0.034	1.83 (0.85-3.90)	0.112
**<16 years**	2.46 (1.04-5.79)	0.040	1.64 (1.01-2.67)	0.047	1.54 (0.21-11.14)	0.67	2.01 (0.67-6.05)	0.200
Smoking Pack-year [10 pack year]*	1.03 (0.96-1.10)	0.476	1.07 (1.01-1.12)	0.02	0.97 (0.64-1.48)	0.901	1.02 (0.93-1.12)	0.606
**Age (Years)**	1.05 (1.04-1.07)	<.0001	1.04 (1.03-1.05)	<.0001	1.05 (1.04-1.07)	<.0001	1.03 (1.02-1.05)	0.001
**BMI (kg/m**^**2**^**)**	1.09 (1.04-1.15)	0.001	1.08 (1.05-1.11)	<.0001	1.11 (1.07-1.16)	<.0001	1.08 (1.06-1.10)	<.0001
**Family Income Percentile**								
**<25%**	1.00		1.00		1.00		1.00	
**26%–50%**	0.72 (0.43-1.21)	0.215	1.05 (0.72-1.55)	0.777	1.35 (0.86-2.11)	0.197	1.07 (0.71-1.62)	0.719
**51%–75%**	0.68 (0.38-1.25)	0.213	0.86 (0.49-1.50)	0.568	1.21 (0.68-2.15)	0.511	0.71 (0.42-1.23)	0.205
**>75%**	0.76 (0.44-1.31)	0.323	0.73 (0.45-1.21)	0.203	0.76 (0.42-1.39)	0.375	0.85 (0.42-1.70)	0.616
**Education**								
**Less than Elementary School**	1.00		1.00		1.00		1.00	
**Less than Middle School**	1.45 (0.88-2.40)	0.144	0.84 (0.46-1.54)	0.555	0.67 (0.35-1.27)	0.218	0.73 (0.32-1.64)	0.418
**High School Diploma**	1.26 (0.78-2.05)	0.346	0.87 (0.53-1.42)	0.556	1.00 (0.60-1.68)	0.987	0.51 (0.25-1.02)	0.056
**At Least Some College**	0.86 (0.47-1.58)	0.625	0.89 (0.58-1.35)	0.557	0.61 (0.22-1.72)	0.349	0.44 (0.23-0.83)	0.014
**Marital Status**								
**Married**	1.00		1.00		1.00		1.00	
**Other**	0.86 (0.46-1.63)	0.647	1.11 (0.78-1.56)	0.545	0.96 (0.68-1.36)	0.821	0.84 (0.61-1.16)	0.271
**Alcohol Consumption**								
**Not At All**	1.00		1.00		1.00		1.00	
**Less than Once a Month**	1.39 (0.81-2.39)	0.235	0.92 (0.66-1.29)	0.602	0.95 (0.65-1.38)	0.773	0.51 (0.30-0.87)	0.017
**More than Twice a Month**	1.05 (0.66-1.66)	0.852	0.73 (0.21-2.53)	0.603	0.91 (0.55-1.50)	0.704	0.94 (0.34-2.60)	0.893
**Moderate Intensity Activity**								
**Yes**	1.00		1.00		1.00		1.00	
**No**	0.98 (0.67-1.43)	0.917	1.15 (0.74-1.77)	0.52	1.24 (0.89-1.71)	0.198	1.26 (0.80-1.98)	0.305
Daily Energy Intake (kcal)*	1.00 (1.00-1.00)	0.018	1.00 (1.00-1.00)	0.142	1.00 (1.00-1.00)	0.459	1.00 (1.00-1.00)	0.215
**Race**								
**Non-Hispanic White**			1				1	
**Mexican American**			1.70 (1.01-2.87)	0.048			1.37 (0.62-3.05)	0.414
**Other Hispanic**			2.35 (1.17-4.70)	0.019			2.08 (1.18-3.67)	0.015
**Non-Hispanic Black**			2.03 (1.20-3.44)	0.011			1.87 (1.12-3.12)	0.019
**Other Race (Including Multiracial)**			1.86 (0.78-4.42)	0.149			3.46 (1.44-8.30)	0.008
**Estimated number of Population**	***23,681,690***		***84,085,965***		***23,193,263***		***78,989,240***	

This study also found that incidence of type II diabetes was significantly associated with age and BMI (in all groups) but not family income, education level, marital status, moderate physical activity, alcohol consumption, or daily energy intake. However, racial disparities among U.S. participants were observed. Hispanic participants (excluding Mexican Americans) had an increased risk of type II diabetes compared with the reference group (non-Hispanic whites) for both men and women (men: HR 2.35, 95% CI 1.17–4.70; women: HR 2.08, 95% CI 1.18–3.67). A similar trend was observed among non-Hispanic blacks (men: HR 2.03, 95% CI 1.20–3.44; women: HR 1.87, 95% CI 1.12–3.12). Among U.S. women, the category “other races” (including multiracial participants) showed a higher risk of type II diabetes (HR 3.45, 95% CI 1.45–8.22) compared with non-Hispanic whites. Finally Mexican American men had a higher risk of type II diabetes than non-Hispanic white men (HR 1.70, 95% CI 1.01–2.87).

Because many observations on SES and life-style variables were lacking, we additionally conducted a robustness check on the analysis of U.S. participants. Variables that were included in this analysis was smoking onset age, smoking pack-year, age, BMI, family income percentile, and race. The results confirmed that early onset and pack-years of smoking were risk factors for type II diabetes for U.S. men population. Furthermore, ages, BMI, race of both sexes were also significant in this robustness check (Table 4).

**Table 4:** Robustness check: adjusted hazard ratios for the association between stratified onset age of smoking and diabetes using United States NHANES 2009-2010 with limited # of variables

	**U.S. men**		**U.S. women**	
	***Hazard ratio***	***P-value***	***Hazard ratio***	***P-value***
	***(95*% *CI)***		***(95*% *CI)***	
**Smoking Onset Age**				
**Never Smoked**	1.00		1.00	
**≥26 years**	1.04 (0.55-1.98)	0.887	1.23 (0.63-2.44)	0.522
**21–25 years**	1.36 (0.66-2.79)	0.381	0.81 (0.44-1.55)	0.520
**16–20 years**	1.38 (1.00-1.91)	0.050	1.39 (0.95-2.05)	0.082
**<16 years**	1.49 (1.08-2.06)	0.018	1.67 (0.87-3.23)	0.117
Smoking Pack-year [10 pack year]*	1.08 (1.02-1.14)	0.001	1.03 (0.95-1.13)	0.508
**Age (Years)**	1.06 (1.06-1.08)	<.0001	1.07 (1.06-1.08)	<.0001
**BMI (kg/m**^**2**^**)**	1.10 (1.07-1.13)	<.0001	1.08 (1.07-1.10)	<.0001
**Family Income Percentile**				
**<25%**	1.00		1.00	
**26%–50%**	1.03 (0.72-1.47)	0.843	1.22 (0.89-1.68)	0.207
**51%–75%**	0.98 (0.60-1.59)	0.925	0.87 (0.52-1.46)	0.570
**>75%**	0.93 (0.70-1.24)	0.611	0.81 (0.42-1.56)	0.503
**Race**				
**Non-Hispanic White**	1		1	
**Mexican American**	1.97 (1.25-3.11)	0.006	2.48 (1.76-3.49)	<.0001
**Other Hispanic**	2.16 (1.36-3.57)	0.005	2.63 (1.39-4.95)	0.005
**Non-Hispanic Black**	1.89 (1.19-3.02)	0.010	1.96 (1.36-2.83)	0.001
**Other Race (Including Multiracial)**	2.96 (1.82-4.79)	<.0001	3.91 (2.13-7.19)	<.0001
**N**	***4,747***		***4,783***	
**Estimated number of Population**	***139,060,000***		***144,740,000***	

## Discussion

Using data from a nationally representative sample of participants from South Korea and the U.S. this study found evidence that early onset of smoking increases the risk of type II diabetes in men of both nations, after adjusting for many confounders. Other studies have reported that the prevalence of type II diabetes among South Korea adults is 7.9%, which is slightly above the average reported by the Organization for Economic Co-operation and Development [39]. The prevalence of type 2 diabetes among U.S. adults in 2010 has been reported as 11.3% (men: 11.8%, women: 10.8%) [8]. Although the prevalence of type II diabetes in this study appeared to be slightly lower for South Korea and higher for the U.S. compared with other sources, our results are fairly consistent with the estimated prevalence in others.

The risk on type II diabetes was greatest and statistically significant among participants who started smoking before age 16 (for both nations), and smoking onset from 16 to 20 years also increased the risk of type II diabetes among U.S. men. The robustness check which included more individuals without few variables also confirmed that the association. Previous studies reported that the risk of type II diabetes increased with higher pack-years of smoking. This was also observed among U.S. men in our study, but this phenomenon did not generalize to the other sample populations. A large cohort study conducted in South Korea [29] identified smoking amount as a risk factor for type II diabetes but did not observe an association with smoking duration. This question may remain unanswered because of the underlying mechanisms of smoking (biological, behavioral) or differences in race or diet. Further study should be conducted to investigate possible explanations for whether the differences are due to clinical or/and behavioral factors. This study also confirmed that age and BMI are important risk factors for type II diabetes, as prior studies have consistently demonstrated [9,10,17,18].

For the U.S. specifically, this study confirms that racial disparities exist in the prevalence of type II diabetes, which is higher in Hispanic and black populations than in white populations. Results of this study may be relevant for policies regarding tobacco use. Many current or former male smokers in South Korea and the U.S. began smoking before the age of 16 or 20 years, which is not legal in either country. Underage smoking may increase the risk of type II diabetes; therefore, strong policies and punishments for selling tobacco to underage youth are needed. The need for actions and interventions to prevent adolescents from smoking is not only given by the risk for type II diabetes but also by the risk of other smoking related diseases as well. In addition, educational programs are needed in schools, especially for male adolescents, and community health programs and incentives are needed to control BMI. Also, parental monitoring could play a significant role on adolescent type II diabetes management by influencing direct and indirect effects that promising better health outcome in adolescents with type II diabetes [40].

This study has several limitations worth noting; therefore, caution must be taken when interpreting results and generalizing findings. Although this study used fairly large, nationally representative samples for both counties, which strengthens the generalizability of the results, a significant limitation still remains because of its cross-sectional nature. Because all survey data were collected at the same time, they do not reflect past health behaviors other than the main predicting variables. Important risk factors such as diet, physical activity, and other covariates did not appear to increase the risk of type II diabetes; however, most of the diabetic participants had received a diagnosis before the survey was conducted. Because of this lack of temporality, diabetic participants had a lower daily energy intake. In addition, self-reported survey results do not fully represent participants’ personal behavior and history. To calculate smoking pack-year, this study used the recent smoking habits of the participants, which may not represent their smoking habits throughout the years. Another covariate, physical activity, was also based only on recent behavior. Furthermore, significant missing information, mainly socioeconomic status covariates of U.S. population might be another limitation on our study. As stated, this study excluded participants who received a diagnosis before age 20 in an attempt to include only those with type II diabetes. Further study should be conducted by including type I diabetic participants and investigate risk differences for each type of diabetes.

Another potential limitation of this study is the false response of smoking status by South Korean women. As shown in Table 1, 4% of the South Korean women (and only 1% of the diabetic participants) reported a history of smoking, but this does not represent the actual smoking rate. Confucianism, which is deeply rooted in the Korean society, holds a negative view of female smokers; therefore, these respondents may have chosen not to report their smoking status, even though the survey was anonymous. This reliance on self-reported cross-sectional information without verification may have caused underreporting of the actual smoking status in all groups.

Although this study is based on cross-sectional and observational data, we believe that this is the one of few comparative investigations to evaluate type II diabetes risk in relation to combination of onset age of smoking and pack-years using nationally representative survey data of both nations which might enhance generality of the association. Our findings deliver public health awareness, especially for adolescents. Many current and former smokers started smoking during adolescence [37] and these smokers are more likely to be current smokers [38]. Early onset of smoking increases the risk for type II diabetes and other smoking-related diseases. Our findings add to the mounting evidences of an association between smoking and type II diabetes, and are relevant for both South Korea and the U.S. Our findings enhance current data on the association between smoking and type II diabetes and are the basis for controlling the illegal sale of cigarettes to underage youth as well as cigarette use. Successful evidences for the enhanced effects of European countries are available regarding tobacco control policies, educational programs, and other behavioral, legislative activities [41,42]. In order to strengthen the reliability and generalizability of these findings, further study is needed using large cohorts and long follow-up periods. Additionally, clinical research is also required to examine the biological drivers of early onset of smoking.

## Conclusion

This study found evidences that early onset of smoking increases type II diabetes risk among men in South Korea and the U.S., and type II diabetes risk increases with higher pack-years in U.S. men. Underage smoking may increase the risk other smoking-related diseases, thus not only education programs but also strong tobacco policies and punishments for selling tobacco to underage youth should be implemented in both South Korea and United State.

## Competing interest

No conflicts of interest relevant to this article and were reported.

## Authors’ contributions

SJK designed the study, researched data, performed statistical analyses, and wrote the manuscript. ECP designed the study, contributed to discussion, and reviewed and edited the manuscript. SHJ and JMN provided recommendations regarding statistical analysis, contributed to discussion, and reviewed and edited the manuscript. WHC and JHK provided recommendations and edited the manuscript. ECP is the guarantor of this work and, as such, had full access to all the data in the study and takes responsibility for the integrity of the data and the accuracy of the data analysis. All authors read and approved the final manuscript.

## Pre-publication history

The pre-publication history for this paper can be accessed here:

http://www.biomedcentral.com/1471-2458/14/178/prepub

## References

[B1] Danaei G Finucane MM Lu Y Singh GM Cowan MJ Paciorek CJ Lin JK Farzadfar F Khang YH Stevens GA National, regional, and global trends in fasting plasma glucose and diabetes prevalence since 1980: systematic analysis of health examination surveys and epidemiological studies with 370 country-years and 2.7 million participants Lancet 2011 378 9785 31 40 10.1016/S0140-6736(11)60679-X 21705069

[B2] King H Aubert RE Herman WH Global burden of diabetes, 1995-2025: prevalence, numerical estimates, and projections Diabetes Care 1998 21 9 1414 1431 10.2337/diacare.21.9.1414 9727886

[B3] Mokdad AH Ford ES Bowman BA Nelson DE Engelgau MM Vinicor F Marks JS The continuing increase of diabetes in the US Diabetes Care 2001 24 2 412 10.2337/diacare.24.2.412 11213906

[B4] Park Y Lee H Koh CS Min H Yoo K Kim Y Shin Y Prevalence of diabetes and IGT in Yonchon County, South Korea Diabetes Care 1995 18 4 545 548 10.2337/diacare.18.4.545 7497867

[B5] Kim YI Choi CS Kim SW Lee JS Kim HH Lee MS Lee SI Park JY Hong SK Lee KU Prevalence of Diabetes Mellitus and Impaired Glucose Tolerance in Korean Adults Living in Jungup District South Korea J Korean Diabetes Assoc 1998 22 3 363 371

[B6] Statistics Korea Causes of Death Statistics in 2011 2011 http://kostat.go.kr/portal/english/news/1/1/index.board?bmode=read&aSeq=260590

[B7] Task Force Team for Basic Statistical Study of Korean Diabetes Mellitus Report of task force team for basic statistical study of Korean diabetes mellitus: diabetis in Korea 2007. 1st ed 2008 1 Seoul: Goldfishery

[B8] U.S. Centers for Disease Control and Prevention, National Diabetes Fact Sheet 2011 http://www.cdc.gov/diabetes/pubs/factsheet11.htm

[B9] Mokdad AH Bowman BA Ford ES Vinicor F Marks JS Koplan JP The continuing epidemics of obesity and diabetes in the United States JAMA 2001 286 10 1195 1200 10.1001/jama.286.10.1195 11559264

[B10] Mokdad AH Ford ES Bowman BA Dietz WH Vinicor F Bales VS Marks JS Prevalence of obesity, diabetes, and obesity-related health risk factors, 2001 JAMA 2003 289 1 76 79 12503980 10.1001/jama.289.1.76

[B11] Hilding A Eriksson AK Agardh EE Grill V Ahlbom A Efendic S Ostenson CG The impact of family history of diabetes and lifestyle factors on abnormal glucose regulation in middle-aged Swedish men and women Diabetologia 2006 49 11 2589 2598 10.1007/s00125-006-0402-5 16969647

[B12] Perez Gomez G Huffman FG Risk factors for type 2 diabetes and cardiovascular diseases in Hispanic adolescents J Adolesc Health 2008 43 5 444 450 10.1016/j.jadohealth.2008.03.010 18848672

[B13] Jeon CY Lokken RP Hu FB van Dam RM Physical activity of moderate intensity and risk of type 2 diabetes: a systematic review Diabetes Care 2007 30 3 744 752 10.2337/dc06-1842 17327354

[B14] Kriska AM Saremi A Hanson RL Bennett PH Kobes S Williams DE Knowler WC Physical activity, obesity, and the incidence of type 2 diabetes in a high-risk population Am J Epidemiol 2003 158 7 669 675 10.1093/aje/kwg191 14507603

[B15] Hu FB Manson JE Stampfer MJ Colditz G Liu S Solomon CG Willett WC Diet, lifestyle, and the risk of type 2 diabetes mellitus in women New Engl J Med 2001 345 11 790 797 10.1056/NEJMoa010492 11556298

[B16] Parillo M Riccardi G Diet composition and the risk of type 2 diabetes: epidemiological and clinical evidence Br J Nutr 2004 92 1 7 19 10.1079/BJN20041117 15230984

[B17] U. S. Department of Health and Human Services National Diabetes Information Clearinghouse(NDIC), Risk factors for type 2 diabetes 2012 http://diabetes.niddk.nih.gov/dm/pubs/riskfortype2/index.aspx

[B18] Yang W Lu J Weng J Jia W Ji L Xiao J Shan Z Liu J Tian H Ji Q Zhu Q Ge J Lin L Chen L Guo X Zhao Z Li Q Zhou Z Shan G He J Prevalence of diabetes among men and women in China New Engl J Med 2010 362 12 1090 1101 10.1056/NEJMoa0908292 20335585

[B19] Facchini FS Hollenbeck CB Jeppesen J Chen YD Reaven GM Insulin resistance and cigarette smoking Lancet 1992 339 8802 1128 1130 10.1016/0140-6736(92)90730-Q 1349365

[B20] Manson JE Ajani UA Liu S Nathan DM Hennekens CH A prospective study of cigarette smoking and the incidence of diabetes mellitus among US male physicians Am J Med 2000 109 7 538 542 10.1016/S0002-9343(00)00568-4 11063954

[B21] Rimm EB Chan J Stampfer MJ Colditz GA Willett WC Prospective study of cigarette smoking, alcohol use, and the risk of diabetes in men BMJ (Clinical research ed) 1995 310 6979 555 559 10.1136/bmj.310.6979.555 7888928 PMC2548937

[B22] Ronnemaa T Ronnemaa EM Puukka P Pyorala K Laakso M Smoking is independently associated with high plasma insulin levels in nondiabetic men Diabetes Care 1996 19 11 1229 1232 10.2337/diacare.19.11.1229 8908385

[B23] Wannamethee SG Shaper AG Perry IJ Smoking as a modifiable risk factor for type 2 diabetes in middle-aged men Diabetes Care 2001 24 9 1590 1595 10.2337/diacare.24.9.1590 11522704

[B24] Carlsson S Midthjell K Grill V Smoking is associated with an increased risk of type 2 diabetes but a decreased risk of autoimmune diabetes in adults: an 11-year follow-up of incidence of diabetes in the Nord-Trondelag study Diabetologia 2004 47 11 1953 1956 10.1007/s00125-004-1554-9 15558231

[B25] Meisinger C Doring A Thorand B Lowel H Association of cigarette smoking and tar and nicotine intake with development of type 2 diabetes mellitus in men and women from the general population: the MONICA/KORA Augsburg Cohort Study Diabetologia 2006 49 8 1770 1776 10.1007/s00125-006-0298-0 16710672

[B26] Foy CG Bell RA Farmer DF Goff DC Jr Wagenknecht LE Smoking and incidence of diabetes among U.S. adults: findings from the Insulin Resistance Atherosclerosis Study Diabetes Care 2005 28 10 2501 2507 10.2337/diacare.28.10.2501 16186287

[B27] Will JC Galuska DA Ford ES Mokdad A Calle EE Cigarette smoking and diabetes mellitus: evidence of a positive association from a large prospective cohort study Int J Epidemiol 2001 30 3 540 546 10.1093/ije/30.3.540 11416080

[B28] Willi C Bodenmann P Ghali WA Faris PD Cornuz J Active smoking and the risk of type 2 diabetes: a systematic review and meta-analysis JAMA 2007 298 22 2654 2664 10.1001/jama.298.22.2654 18073361

[B29] Jee SH Foong AW Hur NW Samet JM Smoking and risk for diabetes incidence and mortality in Korean men and women Diabetes Care 2010 33 12 2567 2572 10.2337/dc10-0261 20823342 PMC2992192

[B30] Cho NH Chan JC Jang HC Lim S Kim HL Choi SH Cigarette smoking is an independent risk factor for type 2 diabetes: a four-year community-based prospective study Clin Endocrinol 2009 71 5 679 685 10.1111/j.1365-2265.2009.03586.x 19508609

[B31] Rafalson L Donahue RP Dmochowski J Rejman K Dorn J Trevisan M Cigarette smoking is associated with conversion from normoglycemia to impaired fasting glucose: the Western New York Health Study Ann Epidemiol 2009 19 6 365 371 10.1016/j.annepidem.2009.01.013 19345115 PMC2723860

[B32] Oh SW Yoon YS Lee ES Kim WK Park C Lee S Jeong EK Yoo T Association between cigarette smoking and metabolic syndrome: the Korea National Health and Nutrition Examination Survey Diabetes Care 2005 28 8 2064 2066 10.2337/diacare.28.8.2064 16043763

[B33] Al-Delaimy WK Willett WC Manson JE Speizer FE Hu FB Smoking and mortality among women with type 2 diabetes: The Nurses’ Health Study cohort Diabetes Care 2001 24 12 2043 2048 10.2337/diacare.24.12.2043 11723080

[B34] Yeh HC Duncan BB Schmidt MI Wang NY Brancati FL Smoking, Smoking Cessation, and Risk for Type 2 Diabetes MellitusA Cohort Study Ann Intern Med 2010 152 1 10 17 10.7326/0003-4819-152-1-201001050-00005 20048267 PMC5726255

[B35] Luo J Rossouw J Tong E Giovino GA Lee CC Chen C Ockene JK Qi L Margolis KL Smoking and Diabetes: Does the Increased Risk Ever Go Away? Am J Epidemiol 2013 178 6 937 945 10.1093/aje/kwt071 23817918 PMC3816526

[B36] Zhang L Curhan GC Hu FB Rimm EB Forman JP Association between passive and active smoking and incident type 2 diabetes in women Diabetes Care 2011 34 4 892 897 10.2337/dc10-2087 21355099 PMC3064047

[B37] Viswanath K Herbst RS Land SR Leischow SJ Shields PG Tobacco and cancer: an American Association for Cancer Research policy statement Canc Res 2010 70 9 3419 3430 10.1158/0008-5472.CAN-10-1087 20388799

[B38] U.S. Centers for Disease Control and Prevention Preventing tobacco use among young people: a report of the Surgeon General 1994 http://www.cdc.gov/tobacco/data_statistics/sgr/1994/index.htm 22876391

[B39] OECD Health at a Glance 2011 2011 http://www.oecd-ilibrary.org/social-issues-migration-health/health-at-a-glance-2011/diabetes-prevalence-and-incidence_health_glance-2011-13-en

[B40] Ellis DA Templin TN Podolski CL Frey MA Naar-King S Moltz K The parental monitoring of diabetes care scale: development, reliability and validity of a scale to evaluate parental supervision of adolescent illness management J Adolesc Health 2008 42 2 146 153 10.1016/j.jadohealth.2007.08.012 18207092

[B41] Schaap MM Kunst AE Leinsalu M Regidor E Ekholm O Dzurova D Helmert U Klumbiene J Santana P Mackenbach JP Effect of nationwide tobacco control policies on smoking cessation in high and low educated groups in 18 European countries Tob Control 2008 17 4 248 255 10.1136/tc.2007.024265 18483129

[B42] Martínez-Sánchez JM Fernández E Fu M Gallus S Martínez C Sureda X La Vecchia C Clancy L Smoking behaviour, involuntary smoking, attitudes towards smoke-free legislations, and tobacco control activities in the European Union PloS One 2010 5 11 e13881 10.1371/journal.pone.0013881 21079729 PMC2975630

